# Recipients of 2023 CSSA Editor's Citation for Excellence named

**DOI:** 10.1002/tpg2.20458

**Published:** 2024-04-22

The editorial board of *The Plant Genome* is pleased to announce the recipients of the 2023 Editor's Citation for Excellence. These awards recognize the outstanding professional commitment and dedication of volunteer reviewers and/or editors who, through their excellent insights and comments, have helped maintain the high standard and quality of papers published in the journal. Recipients were nominated based on their thorough, competent, and timely reviews or editing of manuscripts. Each will receive a certificate of appreciation, an ASA‐CSSA‐SSSA gift certificate, and recognition in CSA News.

## ASSOCIATE EDITORS


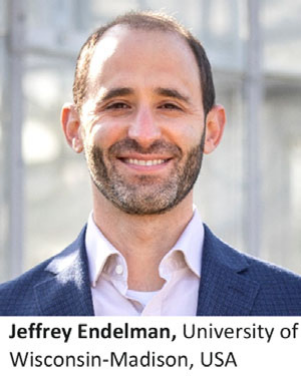
Dr. Jeff Endelman is an Associate Professor at the University of Wisconsin (UW)‐Madison, and a member of *The Plant Genome* editorial board since 2019. He is the director of the UW potato breeding program and conducts both theoretical and applied research in quantitative genetics and genomics. Dr. Endelman has created multiple software packages used in over 1000 studies, with a focus on polyploids. He is also active in the worldwide effort to create diploid, inbred lines of potato for hybrid variety development.


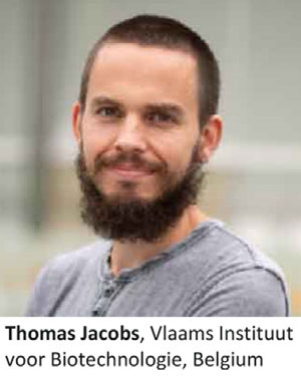
Dr. Thomas Jacobs obtained his PhD from the University of Georgia with Wayne Parrott, developing several molecular tools for soybean biotechnology. In 2014, he moved to the Boyce Thompson Institute for a postdoc with Greg Martin. There, he started developing CRISPR screens in plants. In 2016, he started the Plant Genome Editing group at the VIB‐UGent Center for Plant Systems Biology. In 2022, he was appointed Associate Professor at the University of Gent. His group develops easy‐to‐use genome editing systems for all kinds of plant species, specifically focused on making large‐scale gene knockouts and base editing.


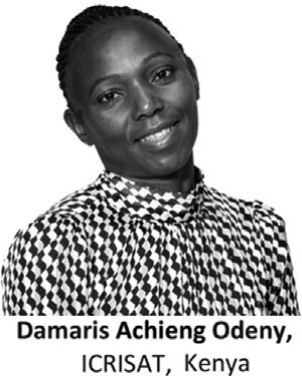
Dr. Damaris Odeny is a plant molecular breeder with global research experience cutting across Africa, Asia, Europe, and United States. She has led and implemented genomics projects in both cereals and legumes, including the development of genomic resources in several underutilized crops. Dr. Odeny earned her PhD in Plant Genetics from the University of Bonn (Germany) and completed a post‐doctoral training from the Max Planck Institute for Plant Breeding Research in Cologne, Germany.

## REVIEWERS


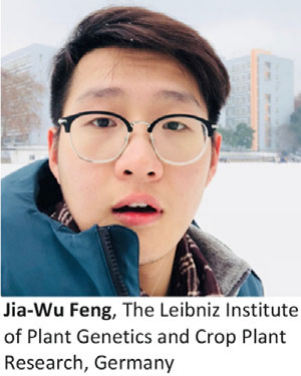
Jia‐Wu Feng is a dedicated bioinformatics researcher with a passion for plant genomes. Currently pursuing a PhD at the Leibniz Institute of Plant Genetics and Crop Plant Research (IPK) in Germany, he is deeply involved in the genomic analysis of bulbous barley and barley's wild relatives, aiming to uncover valuable insights into crop evolution and adaptation. Jia‐Wu Feng's expertise in genome analysis, transcriptomics, and molecular biology addresses key questions in plant biology and contributes to our understanding of crop improvement and adaptation.


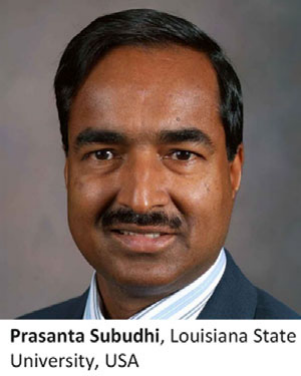
Dr. Prasanta Subudhi is a professor of genetics and genomics at the Louisiana State University Agricultural Center. He has more than thirty years of experience focusing on improving abiotic stress tolerance in cereal crops such as rice and sorghum. He has demonstrated an unwavering commitment to advancing agricultural innovation, sustainability, and global food security. Currently, he is leading a USDA‐National Institute of Food and Agriculture–funded transdisciplinary multi‐institutional coordinated agricultural project with the goal of improving the sustainability of rice farming system in the rice‐growing states of the southern United States.


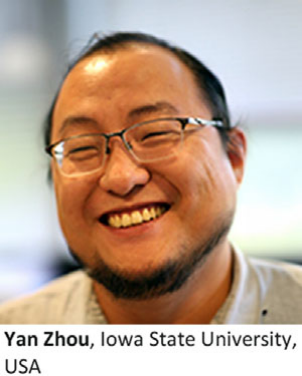
Dr. Yan Zhou is currently working as postdoctoral research associate at Iowa State University (Dr. Patrick Schnable's lab). His work focuses on developing high‐throughput image‐based phenotyping pipelines and using different strategies to dissect the genetic factors altering plant architectures in maize and sorghum. He obtained his MS degree in Genetics from Nanjing Agricultural University, China. He later joined industry, switched from wheat to clone male sterile genes in maize, and helped develop a hybrid seed production system using a transgenic multicontrol sterility system. He earned his PhD from Iowa State University focusing on applying integrated phenomic approaches to study the genetic basis of inflorescence and leaf in grain crops.

